# A Synopsis or Systematic Catalogue of the Medical Plants of the United States

**Published:** 1853-05

**Authors:** 


### EDITORIAL.

*Synopsis or Systematic Catalogue of the Medicinal Plants of the United States.* By A. Clapp, M. D. Presented to the American Medical Association, at its Session of May, 1852; and published in the 5th vol. of its transactions. Philadelphia: T. K. and P. G. Collins, 1852. From the Author.

The author has followed in his arrangement of orders, genera and species, Dr. Gray's *Manual of the Botany of the Northern United States.*

Dr. Clapp is a resident of New Albany, Ind., and has devoted much of his time, for the last twenty years, to the study of his favorite science. He has evidently bestowed on this synopsis a large amount of labor. The medical references are mostly American. The work is marred by numerous typographical errors, which, in our copy, has been carefully corrected by the author himself. The catalogue embraces both our indigenous and naturalized plants, and is a valuable addition to our medical literature.

We learned recently that the laurus camphorus has been introduced into Louisiana. The climate and soil are believed to be adapted to its culture, and the experiment so far has been successful. There is also a species of Aristolochia, the wauaco, a native of Mexico, but which has been cultivated on the plantation of Ex-Governor Roman, of La., said to be a specific against the bite of a rattle snake. Experiments have been performed by M. Contois, on dogs and other animals; those treated with the aristolochia recovered speedily, the others died, apparently in great distress. Two negroes were bitten on the same plantation, and were cured by the same means. We mention these facts simply to call the attention of the profession to the adaptation of our soil and climate to foreign medicinal plants. Between Maine and Texas, and the Atlantic and Pacific, we have the soil, temperature, and moisture, of almost all lands. Why, then, may we not produce our own vegetable medicines, at least to a great extent, thereby protecting ourselves against the imposition of adulterated drugs?

We would also earnestly request the profession in the North-West to cultivate our native medical botany, and especially to investigate the properties of those plants reputed to possess medicinal virtue. The records of the profession are meager, and our knowledge very imperfect. We fear that the appropriation of botanic medicines by a certain class of charlatans, has deterred many from making a judicious use of them, lest they might be ranked in the same category. J.

